# Crosswalk between the PROMIS physical function CAT and PROMIS upper extremity CAT v1.2 in a hand surgery population

**DOI:** 10.1186/s41687-024-00736-6

**Published:** 2024-05-30

**Authors:** Miranda J. Rogers, Joshua R. Daryoush, Chong Zhang, Amy Cizik, Angela P. Presson, Nikolas H. Kazmers

**Affiliations:** 1 Department of Orthopaedic Surgery, University of Michigan, Ann Arbor, MI 48109 USA; 2 Department of Orthopaedic Surgery, University of Utah, 590 Wakara Way, Salt Lake City, UT 84108 USA; 3 Division of Public Health, University of Utah, Salt Lake City, UT 84108 USA

**Keywords:** Crosswalk study, Computer adaptive testing, Patient-reported outcome measures, Patient-reported outcomes measurement information system, Physical function, PROM, PROMIS-PF, PROMIS-UE CAT, Upper extremity

## Abstract

**Background:**

There is no gold standard patient-reported outcome measure (PROM) in hand surgery. As a result, a diverse array of PROM instruments have been utilized across centers over time. Lack of score interchangeability limits the ability to compare or conglomerate scores when new instruments are introduced. Our aim was to develop a linkage for the PROMIS UE CAT v1.2 and PROMIS PF CAT scores and develop crosswalk tables for interconversion between these PROMs.

**Methods:**

Retrospective review was conducted to identify adult (≥ 18y) patients seen by orthopaedic hand surgeons at a single academic tertiary care hospital who had completed PROMIS UE CAT v1.2 and PROMIS PF CAT score at the same visit. For those with multiple visits, only one randomly selected visit was included in the analyses. Pearson’s correlation was calculated to determine the linear relationship between the scores. Linkage from PF to UE was performed utilizing several commonly utilized equating models (identity, mean, linear, equipercentile and circle-arc methods). The performance of the models was assessed using intraclass correlation (ICC) between observed PROMIS UE CAT v1.2 and estimated PROMIS UE CAT v1.2 scores generated using the model as well as Root Mean Square Error (RMSE). The model chosen as the ‘best’ was further assessed for population invariance using root expected mean squared difference (REMSD) where < 0.08 were considered good.

**Results:**

Of 10,081 included patients, mean age was 48.3 (SD = 17.0), and 54% were female (5,477/10,081). Mean UE CAT v1.2 and PF CAT scores were 37 (SD = 9.8) and 46 (SD = 10.0), respectively. There was a strong correlation between the scores (Pearson correlation *r* = 0.70). All methods performed acceptably (ICC ≥ 0.66 and RMSE < = 7.52 for all). The equipercentile method had the highest ICC (ICC = 0.70 (95% CI 0.69–0.71)) while the mean and circle arc methods had the lowest RMSE. The circle arc method is the most reliable with the smallest standard error and has satisfactory population invariance across age group (REMSD 0.065) and sex (REMSD 0.036).

**Conclusions:**

Crosswalk tables to be used for bidirectional conversion between scores were created.

**Level of evidence:**

: III.

**Supplementary Information:**

The online version contains supplementary material available at 10.1186/s41687-024-00736-6.

## Background

Patient-reported outcome measures (PROMs) are pre- and post-operative measurements of patient-reported functionality, pain, and quality of life that are utilized in research and clinical practice [1].

In the era of value-based care there is growing emphasis on the collection and reporting of PROMs to facilitate the evaluation of patient conditions and treatment efficacy [2–5]. PROMs provide surgeons with validated measures through which to assess clinical improvement and to compare outcomes with other patient populations or between surgeons. The Patient-Reported Outcomes Measurement Information System (PROMIS) was created to standardize the process of administering and interpreting PROMs [6] and utilizes Computer Adaptive Tests (CAT), which minimizes responder burden [7]. PROMIS metrics are designed with a mean score of 50 and standard deviation (SD) of 10 for the reference population, allowing for easy interpretation [6].

A number of established PROMs are utilized in the field of hand and upper extremity surgery, including PROMIS Physical Function (PROMIS PF) CAT and the PROMIS Upper Extremity (PROMIS UE) CAT, of which the latter has multiple versions—mostly recently version 1.2 and 2.0 [6]. Both PROMIS PF CAT and PROMIS UE CAT have been utilized in the hand and upper extremity population [8–15]. PROMIS UE CAT v1.2 and v2.0 are not interchangeable, but are for PROMIS PF CAT [6, 10]. PROMIS PF CAT provides an overview of overall patient function level without specifically targeting the upper extremity, however, this score has demonstrated validity and responsiveness within hand and upper extremity populations and is ubiquitous throughout literature [16–19]. Much like other PROM instruments, PROMIS PF CAT and PROMIS UE CAT have limitations worth mentioning, including the fact that younger individuals report higher normative PROMIS PF and PROMIS UE scores—indicating greater function—than older individuals [20]. Additionally, there are known concerns regarding floor and ceiling effect for both instruments [6]. A clear consensus on which PROM application and clinical assessment with these measures has not emerged [4, 9, 21–24]. The variety of existing PROMs, lack of universal gold standard, and institutional differences in PROM utilization has led to challenges in directly comparing different groups and research.

Linking represents a mathematical method used to connect two correlated PROM scores, allowing the creation of a common metric. For scores that can be linked, crosswalk tables can be generated to facilitate interconversion. The linking of different PROMs allows for comparison between groups and increases statistical power. It also allows for the inclusion of studies using different PROMs in meta-analyses. Prior studies have shown correlations between the PROMIS PF CAT and PROMIS UE CAT [8, 9], although we are unaware of prior literature that has established a linkage between these two measures. Current literature in our field has emphasized using CAT instruments and it is unclear if short form (SF) CAT scores are interchangeable or not. The study objective was to develop a linkage model between the PROMIS PF CAT score and the PROMIS UE CAT v1.2 score, enabling crosswalks between these frequently utilized PROMs.

## Materials and methods

A retrospective review study approved by the Institutional Review Board was performed including patients from February 2014 to August 2017 who were treated by one of five fellowship-trained orthopaedic hand surgeons at a single academic tertiary care hospital. Patients were included if they had concurrently completed PROMIS UE CAT v1.2 and PROMIS PF CAT instruments at the same clinical encounter. Patients were excluded if they were < 18-year-old or if they were seen for a lower extremity or shoulder primary complaint. PROM instruments were completed by patients in clinic or in the preoperative holding area electronically on a tablet computer. One randomly selected visit was included in the analysis for patients who had multiple visits with concurrent PROMIS PF and PROMIS UE scores.

Total score distribution for both the instruments was summarized descriptively. The linear relationship between the scores was assessed using Pearson correlation. A score between *r* = 0.60–0.79 was considered a “strong relationship” [25, 26] and a score *r* > 0.80 was considered a “very strong relationship.” Linkage between the PROMIS PF CAT and PROMIS UE CAT v1.2 was accomplished using the R package *equate* [27]. Multiple methods were used to develop linkages: identity, mean, linear, equipercentile equating (EE) and circle-arc methods [28, 29]. Standard error and root-mean square errors (RMSE) for the linking functions were assessed using bootstrapping methods, with the total cohort serving as the reference for defining the mean observed difference between observed and estimated scores in these groups. Intraclass correlation coefficients (ICC) was used to assess linkage model performance. The performance and population invariance of the linkage models was further assessed in a subgroup analysis evaluating sex and age (< 60 or ≥ 60-year-old).

## Results

**Fig. 1:**
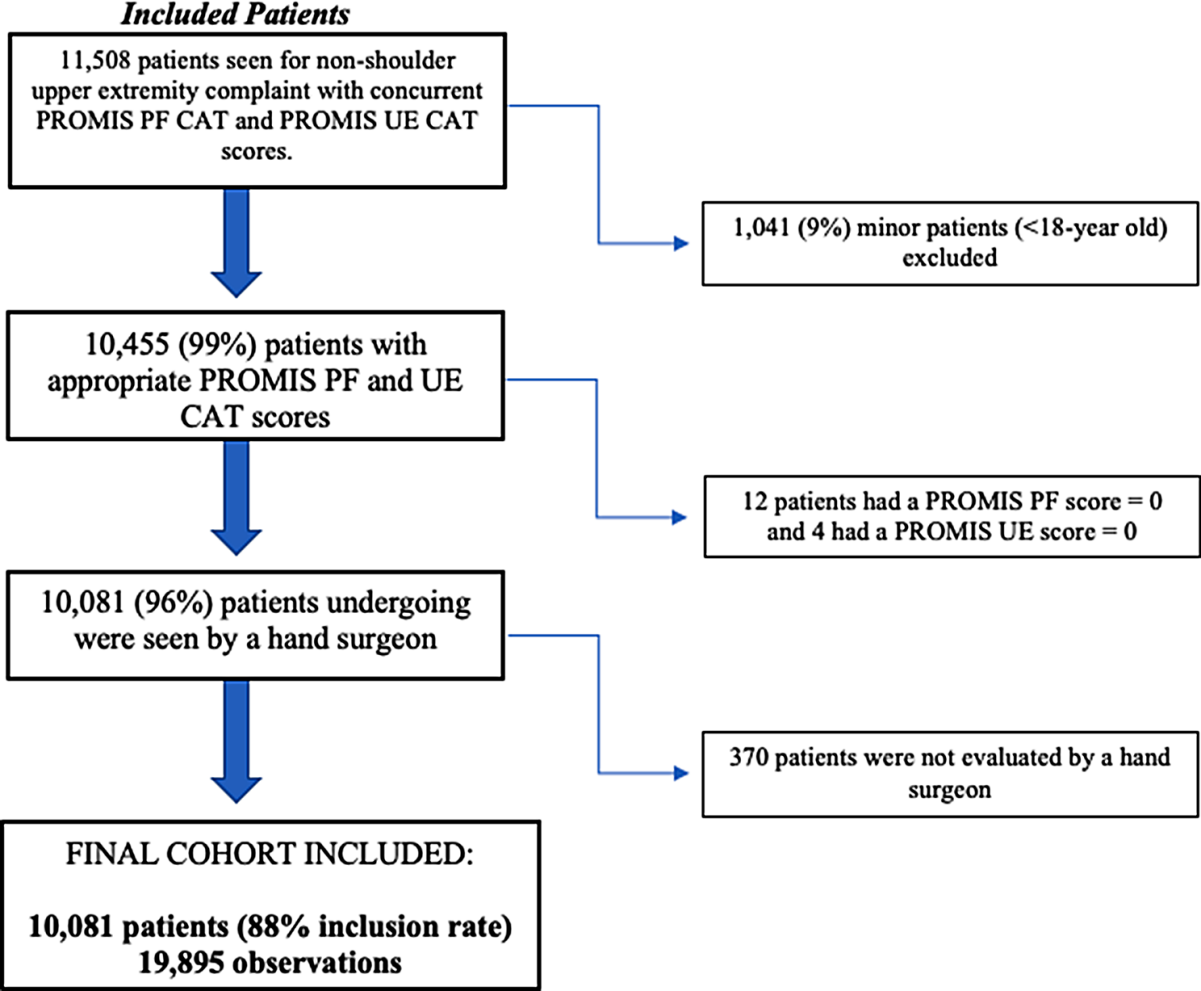
Patient inclusion/exclusion based on study selection criteria

**Fig. 2:**
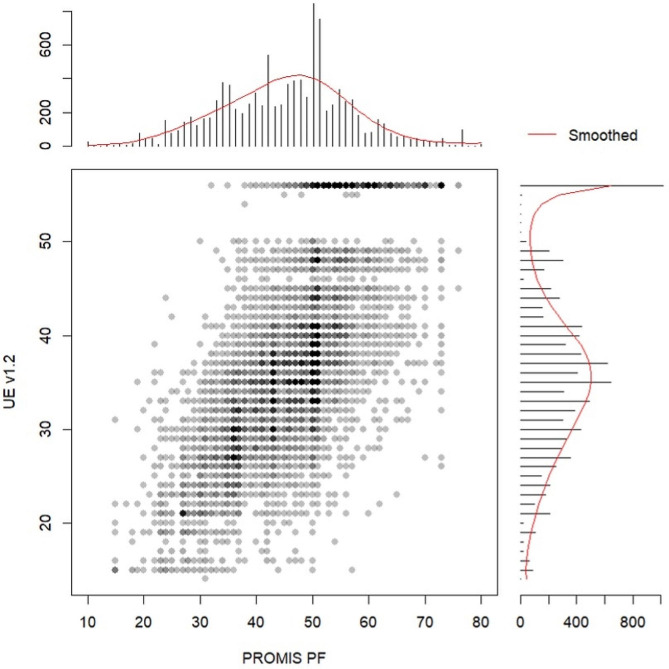
Bivariate and marginal distribution of PROMIS PF CAT and PROMIS UE CAT v1.2 total scores

A total of 11,508 patients were identified who were seen for non-shoulder UE complaint with concurrent PROMIS PF CAT and PROMIS UE CAT v1.2 scores. Minor patients (*n* = 1,041), those with PROMIS PF score = 0 (*n* = 12), those with PROMIS UE score = 0 (*n* = 4), and patients not seen by hand surgeons (*n* = 370) were excluded. Therefore, the final analysis included 10,081 patients (Fig. 1). The mean age was 48 years (SD = 17), 54% were female (5,477/10,081), and 85% were White (8,564/10,081) (Table 1). The mean PROMIS PF CAT score was 46 (SD = 10), and the mean PROMIS UE CAT v1.2 score was 37 (SD = 9.8) (Table 2). A strong positive linear relationship between PROMIS PF CAT and PROMIS UE CAT v1.2 was observed (Pearson’s correlation coefficient *r* = 0.70) (Fig. 2).

**Table 1:** Baseline patient demographics

Variable	*N*/%*
**Age**	
Mean (SD) Median (IQR) Range	48.3 (17.0) 49.0 (34.0, 61.0) (18.0, 102.0)
**Sex**	
Female Male	5477 (54.3%) 4604 (45.7%)
**Race**	
White Black American Indian or Alaska Native Asian Native Hawaiian or Other Pacific Islander Other or did not to disclose	8564 (85.4%) 135 (1.3%) 89 (0.9%) 220 (2.2%) 84 (0.8%) 940 (9.4%)
**Ethnicity**	
Not Hispanic/Latino Hispanic/Latino Did not disclose	9038 (90.2%) 848 (8.5%) 130 (1.3%)
**Marital Status**	
Married Single Divorced Widowed Life Partner/Domestic Partner Legally Separated Other	5803 (58.8%) 2503 (25.3%) 737 (7.5%) 462 (4.7%) 222 (2.2%) 84 (0.9%) 64 (0.6%)
**Surgeon**	
A B C D E	3140 (31.1%) 2537 (25.2%) 2379 (23.6%) 1579 (15.7%) 446 (4.4%)
**Substance Use**	
Active alcohol use Active smoking	3398 (45%) 1054 (10.9%)
**Insurance**	
Commercial Medicare Medicaid Workers’ Compensation Self-Pay Automobile Insurance Other	6634 (65.9%) 2032 (20.2%) 460 (4.6%) 445 (4.4%) 333 (3.3%) 84 (0.8%) 78 (0.8%)

*N = 10,081 patients

*SD* = standard deviation, *IQR* = interquartile range

Missing values: Race = 49, Ethnicity = 65, Marital status = 206, Alcohol use = 2536, Smoking = 437, Insurance = 15

**Table 2:** Summary of PROMIS PF CAT and PROMIS UE CAT v1.2 scores

PROM	Data	Score*
PROMIS PF CAT	Mean (SD) Median (IQR) Range	37 (9.8) 36.0 (30, 42) (14, 56)
PROMIS UE CAT v1.2	Mean (SD) Median (IQR) Range	46 (10) 47 (38, 51) (15, 76)

*N = 10,081

*SD* = standard deviation, *IQR* = interquartile range, *PROMIS* = Patient-Reported Outcomes Measurement Information System, *PF* = physical function, *UE* = upper extremity, *CAT* = Computer Adaptive Tests

**Fig. 3:**
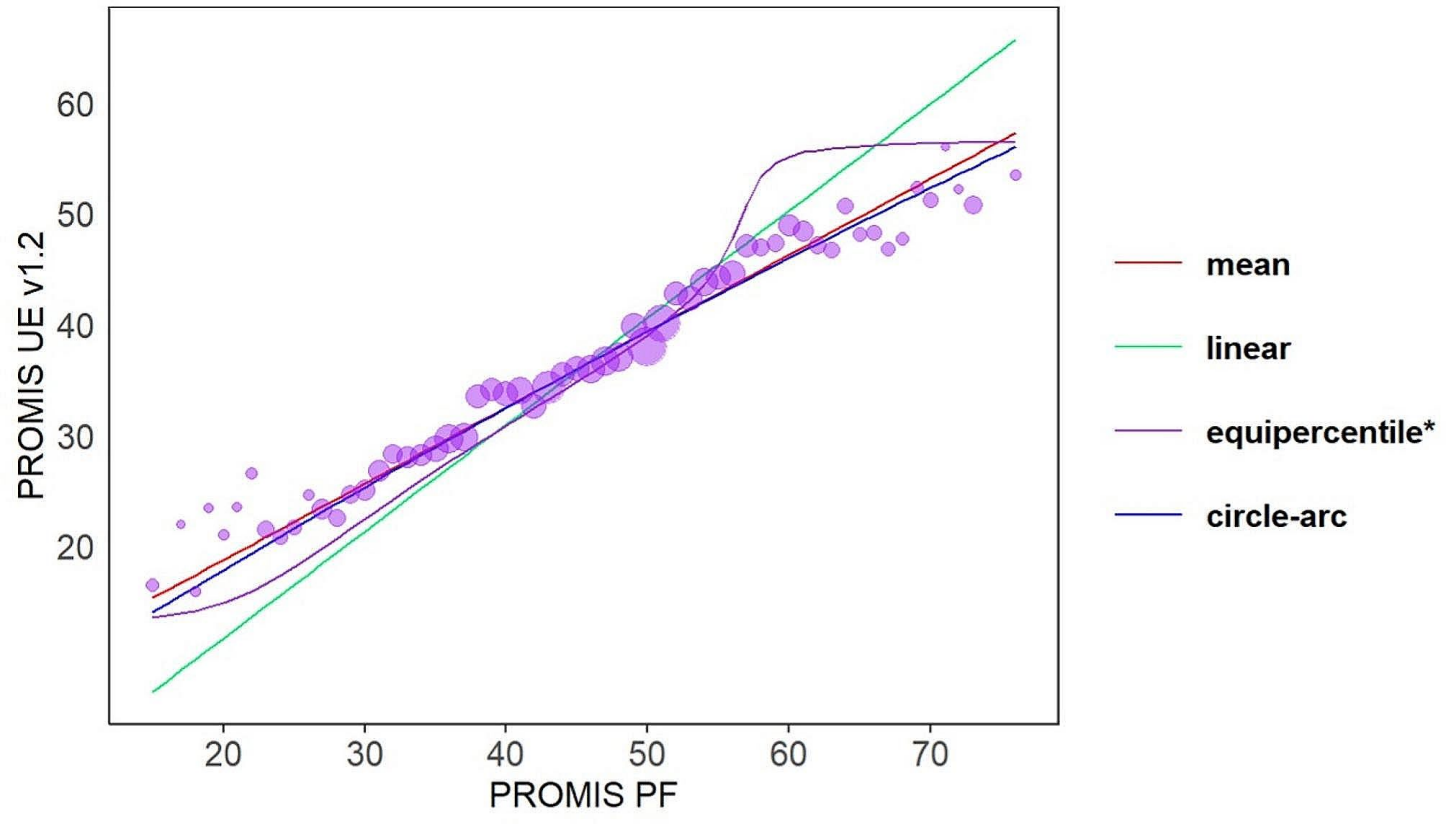
Equating functions by model type versus observed mean PROMIS PF CAT and PROMIS UE CAT v1.2 scores

The various equations for the PROMIS PF CAT and PROMIS UE CAT v1.2 linkage models are displayed in Table 3. All methods performed acceptably (ICC ≥ 0.66 and RMSE < = 7.52 for all) (Fig. 3). The equipercentile method had the highest ICC (ICC = 0.70 (95% CI 0.69–0.71)) while the mean and circle arc methods had the lowest RMSE. The circle arc method is the most reliable with the smallest standard error and has satisfactory population invariance across age group (REMSD 0.065) and sex (REMSD 0.036).

**Table 3:** Performance of the equating models for the PROMIS PF CAT and PROMIS UE CAT v1.2 linkage

Equating Model Type	Formula	ICC	SE	RMSE
Linear regression	y = 5.33 + 0.68x	0.66 (0.65,0.67)	0.13	6.94
Identity	y = 3.67 + 0.69x	0.65 (0.63,0.68)	0	7.09
Mean	y = 5.09 + 0.69x	0.66 (0.65,0.67)	0.07	6.94
Linear	y = -7.87 + 0.97x	0.70 (0.69,0.71)	0.14	7.52
Equipercentile Equating	-	0.70 (0.69,0.71)	0.11	7.44
Circle-arc	-	0.66 (0.65,0.67)	0.05	6.94

*ICC* = Intraclass correlation coefficients, *SE* = standard error, *RMSE* = root mean square error

A crosswalk table linking PROMIS PF CAT to PROMIS UE CAT v1.2 for all linkage models allowing for bidirectional conversion between scores is included in Appendix 1. Table 4 details the application of equipercentile equating model to age and sex subpopulations. ICC, SE, and RMSE were all slightly higher in the subgroups, particularly in the age ≥ 60 population (EE model, RMSE 7.46), (Table 4). However, subgroup analysis by age or sex did not demonstrate significant alteration in equating model performance or introduction of population invariance that would require the production of crosswalk tables dichotomized by age or sex.

**Table 4:** Sex and age subgroup equipercentile linkage model performance for the PROMIS PF CAT and PROMIS UE CAT v1.2 Linkage

Equating model type & Subgroup	#	ICC	SE	RMSE
**Equipercentile equating model**				
Age < 60 Age ≥ 60 Males Females	2967 7114 4604 5477	0.70 (0.68,0.71) 0.71 (0.70,0.72) 0.70 (0.69,0.72) 0.70 (0.68,0.71)	0.18 0.12 0.16 0.14	7.25 7.46 7.66 7.17

*ICC* = Intraclass correlation coefficients, *SE* = standard error, *RMSE* = root mean square error

## Discussion

PROMs are a tool for assessing the results of hand and upper extremity surgery [30] and remain relevant in the clinical setting by capturing the patient’s perception of their own health status, including physical function, social function, quality of life, and other health domains [2, 31]. Given that the proposed use of these instruments includes shared decision-making, quality assurance, post-operative care, the development and interpretation of clinically meaningful research, and value-based care delivery [32], improving our understanding and ability to translate between PROMs is critical for continued research. Our main study finding is that the linking of PROMIS PF CAT and PROMIS UE CAT v1.2 through equating models had acceptable performance. We recommend the use of the equipercentile equating model, due to acceptable performance of the model and frequent utilization of this method in the crosswalk literature [33–37]. This allows for the derivation of bidirectional crosswalk tables that allow for interconversion between PROMIS PF CAT and PROMIS UE CAT v1.2 for future research in the hand and upper extremity population. This improves our ability to combine, synthesize, and understand PROMs in the literature, as well as in meta-analyses including multiple studies with differing methodologies.

The PROMIS UE CAT v1.2 has been administered in many important settings for a range of upper extremity issues, including chronic disease, long-term disability, and acute conditions [11]. Hung et al. documented high correlation between PROMIS UE CAT v1.2 and PROMIS PF CAT, Anxiety CAT, and Pain Interference CAT [11]. They also confirmed that the PROMIS UE CAT v1.2 question bank items represent an independent domain from overall PF. The PROMIS UE CAT will continue to be used in clinical outcomes research and has a role in specifically assessing upper extremity function, noting that the most current version is v2.0 [6]. However, as discussed by Hung et al., the PROMIS UE CAT v1.2 has a ceiling effect that limits its ability to assess patients with higher levels of upper extremity function—seen as a limited number of questions targeted toward those with higher function—that results in an impaired ability to discriminate high-end functioning of the upper extremity [11]. The presence of a ceiling effect is not limited to the PROMIS UE CAT v1.2 and has been documented in the Disabilities of the Arm, Shoulder and Hand questionnaire and Short Musculoskeletal Functional Assessment [17, 38]. Each PROM has its limitations that need to be understood for appropriate instrument application.

It is important to note that the impact of subpopulations (sex and age) on the linkage models was found to be minimal. Across all evaluated groups (male versus female; age < 60 versus age ≥ 60), there were no substantial increased levels of population invariance. These findings suggest a lack of age and sex-specific differences in patient response to these instruments and, as a result, additional crosswalk tables accounting for these subgroup differences were not indicated. This is important, as prior research has documented that younger individuals can have higher normative PROMIS PF and PROMIS UE scores—indicating greater function—than older individuals [20]. As a result, the universal reference of a score of 50 may not apply to certain normative subpopulations [20]. However, given our extensive sample size (> 10,000 patients/>19,000 observations) and broad inclusion (both trauma and elective hand and upper extremity conditions), we contend that our findings hold relevance to populations responsive to the PROMIS PF CAT and PROMIS UE CAT v1.2 measures. Given our findings, the crosswalk tables presented are sufficient for all adult groups (excluding minors) and both sexes.

This study has limitations that warrant mention. The data was collected prospectively but reviewed in a retrospective nature, and not purposely collected for the linking the PROMIS PF CAT to the PROMIS UE CAT v1.2. Retrospective data analysis can introduce confounders, and we did not assess the difference between PROM responders and non-responders. However, the robust PROMs completion rate that our collection workflow yields (> 90%) may reduce this concern [39]. The cohort studied originates from a single institution and comprises predominantly White individuals (85%), which exceeds the general U.S. population by 9.4% [40]. This limits the generalizability of the findings to other populations based on both race/ethnicity and the types of hand and upper extremity pathology seen at the institution. Finally, these results may not apply to shoulder conditions.

## Conclusions

In conclusion, all models equating the PROMIS PF CAT and PROMIS UE CAT v1.2 performed acceptably, although we recommend the use of the equipercentile equating model moving forward. These results allow for crosswalking between PROMIS PF CAT and PROMIS UE CAT v1.2 instruments for adult non-shoulder hand and upper extremity patients with both elective and traumatic diagnoses. Additional crosswalk tables accounting for sex and age were not needed given lack of appreciable population invariance based on these demographic factors. These crosswalk tables have potential applications for development of multicenter clinical databases and broader inclusion in meta-analysis studies, where contributing centers or researchers employ varying PROMs.

### Electronic supplementary material

Below is the link to the electronic supplementary material.

Supplementary Material 1

## Data Availability

The crosswalk tables are available in Appendices I & II submitted with this manuscript. The datasets generated and/or analyzed during the current study are not publicly available due to it being a collected component of patient clinical care but are available from the corresponding author on reasonable request.
