# Supplementary material for: Crosswalk between the PROMIS physical function CAT and PROMIS upper extremity CAT v1.2 in a hand surgery population
Source: J Patient Rep Outcomes. 2024 May 30;8:53. doi: 10.1186/s41687-024-00736-6 (PMC11139816; doi:10.1186/s41687-024-00736-6)
Supplement: Supplementary file 1 — Supplementary Material 1 [file 41687_2024_736_MOESM1_ESM.docx]

**Appendix 1**. Crosswalk Tables for PROMIS PF CAT (15-76) and PROMIS UE CAT (14-56) per identity, mean, linear, equipercentile, and circle-arc models

| **PF Score** | **UE Score** | | | | |
| --- | --- | --- | --- | --- | --- |
|  | **Identity** | **Mean** | **Linear** | **Equipercentile** | **Cirle-arc** |
| 15 | 14 | 15 | 7 | 14 | 14 |
| 16 | 15 | 16 | 8 | 14 | 15 |
| 17 | 15 | 17 | 9 | 14 | 16 |
| 18 | 16 | 17 | 10 | 14 | 16 |
| 19 | 17 | 18 | 11 | 14 | 17 |
| 20 | 17 | 19 | 12 | 15 | 18 |
| 21 | 18 | 19 | 13 | 15 | 19 |
| 22 | 19 | 20 | 14 | 16 | 19 |
| 23 | 20 | 21 | 15 | 17 | 20 |
| 24 | 20 | 21 | 16 | 17 | 21 |
| 25 | 21 | 22 | 16 | 18 | 22 |
| 26 | 22 | 23 | 17 | 19 | 22 |
| 27 | 22 | 24 | 18 | 20 | 23 |
| 28 | 23 | 24 | 19 | 21 | 24 |
| 29 | 24 | 25 | 20 | 22 | 25 |
| 30 | 24 | 26 | 21 | 22 | 25 |
| 31 | 25 | 26 | 22 | 23 | 26 |
| 32 | 26 | 27 | 23 | 24 | 27 |
| 33 | 26 | 28 | 24 | 25 | 27 |
| 34 | 27 | 28 | 25 | 26 | 28 |
| 35 | 28 | 29 | 26 | 27 | 29 |
| 36 | 28 | 30 | 27 | 28 | 30 |
| 37 | 29 | 30 | 28 | 28 | 30 |
| 38 | 30 | 31 | 29 | 29 | 31 |
| 39 | 31 | 32 | 30 | 30 | 32 |
| 40 | 31 | 33 | 31 | 31 | 32 |
| 41 | 32 | 33 | 32 | 32 | 33 |
| 42 | 33 | 34 | 33 | 32 | 34 |
| 43 | 33 | 35 | 34 | 33 | 35 |
| 44 | 34 | 35 | 35 | 34 | 35 |
| 45 | 35 | 36 | 36 | 35 | 36 |
| 46 | 35 | 37 | 37 | 36 | 37 |
| 47 | 36 | 37 | 38 | 36 | 37 |
| 48 | 37 | 38 | 39 | 37 | 38 |
| 49 | 37 | 39 | 40 | 38 | 39 |
| 50 | 38 | 39 | 41 | 39 | 39 |
| 51 | 39 | 40 | 42 | 40 | 40 |
| 52 | 39 | 41 | 43 | 41 | 41 |
| 53 | 40 | 41 | 44 | 42 | 41 |
| 54 | 41 | 42 | 44 | 44 | 42 |
| 55 | 42 | 43 | 45 | 45 | 43 |
| 56 | 42 | 44 | 46 | 48 | 43 |
| 57 | 43 | 44 | 47 | 51 | 44 |
| 58 | 44 | 45 | 48 | 53 | 45 |
| 59 | 44 | 46 | 49 | 55 | 45 |
| 60 | 45 | 46 | 50 | 55 | 46 |
| 61 | 46 | 47 | 51 | 56 | 47 |
| 62 | 46 | 48 | 52 | 56 | 47 |
| 63 | 47 | 48 | 53 | 56 | 48 |
| 64 | 48 | 49 | 54 | 56 | 49 |
| 65 | 48 | 50 | 55 | 56 | 49 |
| 66 | 49 | 50 | 56 | 56 | 50 |
| 67 | 50 | 51 | 57 | 56 | 50 |
| 68 | 50 | 52 | 58 | 56 | 51 |
| 69 | 51 | 52 | 59 | 56 | 52 |
| 70 | 52 | 53 | 60 | 56 | 52 |
| 71 | 53 | 54 | 61 | 56 | 53 |
| 72 | 53 | 55 | 62 | 56 | 54 |
| 73 | 54 | 55 | 63 | 56 | 54 |
| 74 | 55 | 56 | 64 | 56 | 55 |
| 75 | 55 | 57 | 65 | 56 | 55 |
| 76 | 56 | 57 | 66 | 56 | 56 |
| *We recommend using the crosswalk tables derives from the equipercentile equating model for future use. | | | | | |
